# Supplementary material for: Benchmark datasets for SARS-CoV-2 surveillance bioinformatics
Source: PeerJ. 2022 Sep 5;10:e13821. doi: 10.7717/peerj.13821 (PMC9454940; doi:10.7717/peerj.13821)
Supplement: Table S1 [file peerj-10-13821-s001.docx]

Supplementary table 1. GISAID reference accessions.

| VOI/VOC lineage | GISAID reference |
| --- | --- |
| B.1.1.7 | EPI_ISL_1214361 |
| B.1.351 | EPI_ISL_745110 |
| B.1.429 | EPI_ISL_847621 |
| B.1.427 | EPI_ISL_847569 |
| B.1.525 | EPI_ISL_954180 |
| B.1.526 | EPI_ISL_794226 |
| P.1 | EPI_ISL_804824 |
| P.2 | EPI_ISL_717936 |
| B.1.617.1 | EPI_ISL_2088240 |
| B.1.617.2 | EPI_ISL_1718630 |
